# Protocadherin γC4 promotes neuronal survival in the mouse retina through its variable cytoplasmic domain

**DOI:** 10.21203/rs.3.rs-6206977/v1

**Published:** 2025-03-24

**Authors:** Cathy M. McLeod, Hannah G. Lanthier, Garrett R. Nitschke, Samjhana Bhandari, Andrew M. Garrett

**Affiliations:** 1 Department of Pharmacology, Wayne State University School of Medicine, Detroit, Michigan, USA; 2 Department of Ophthalmology, Visual, and Anatomical Sciences, Wayne State University School of Medicine, Detroit, Michigan, USA

**Keywords:** apoptosis, γ-Protocadherin, neural development, retina, mouse

## Abstract

Developmental apoptosis is an important mechanism for the formation of functional neural circuits. Distinct neuronal subtypes undergo apoptosis to a greater or lesser extent during development, although how this is regulated at the cell type level is unknown. The clustered Protocadherins (cPcdhs) are ~60 homophilic cell adhesion molecules expressed from three contiguous gene clusters, which together encode the α-, β-, and γ-Pcdh families. Only one cPcdh isoform, γC4, is essential for survival in the mouse, given its role in attenuating the extent of developmental neuronal apoptosis. However, there is also evidence that other isoforms contribute to neuronal survival. Here we focused on amacrine cell types in the mouse retina, using a series of genetic models to ascertain that γC4 alone accounts for the pro-survival function of the γ-Pcdhs, and that neuronal subtype dependence on γ-Pcdhs for survival correlates with expression of this single isoform. To test which domains of the protein were essential for this function, we employed a rescue approach with *in vitro* live cell imaging, finding that the unique variable cytoplasmic domain of γC4–not its adhesive extracellular cadherin repeats–is necessary and sufficient promote neuronal survival.

## INTRODUCTION

Programmed cell death (PCD) by apoptosis is an essential component of neural development from structural morphogenesis to circuit refinement. PCD in developing neurons must be tightly regulated: Prevention of PCD by mutation of the pro-apoptotic gene *Bax* results in a myriad of neural defects while exacerbation of PCD results in excessive apoptosis and death of the animal (reviewed in [1]. Not all cell types undergo PCD to the same extent. For example, in the cerebral cortex, ~30–40% of differentiated inhibitory interneurons will undergo apoptosis [2, 3] while only ~12% of excitatory projection neurons are lost during development [3]. Cell identity is an important determinant of PCD fate [4], as is neuronal activity, which promotes survival [5, 6]. Thus, PCD can be regulated both by cell intrinsic factors and by a neuron’s success in integrating into a functional circuit.

The γ-Protocadherins (γ-Pcdhs) are a family of 22 homophilic cell adhesion molecules expressed from the *Pcdhg* gene cluster. The *Pcdhg* cluster is arrayed in tandem on mouse chromosome 18 (human chromosome 5q31) with two others – *Pcdha* encodes the 14 α-Pcdhs and *Pcdhb* the 22 β-Pcdhs [7]. Targeted disruption of the *Pcdha* and *Pcdhb* loci – alone or together – had a minimal effect on neuronal survival [8], while mice null for the entire *Pcdhg* cluster (*Pcdhg*^*−/−*^) exhibited extensive neuronal apoptosis and lethality within the first postnatal day [9]. Through use of a conditional allele to circumvent this lethality and investigate later developing structures, it was found that this cell death included interneurons in the spinal cord and cortex [10–12], neurons in the hypothalamus [13], and many neuron types in the retina [14]. It did not, however, include lower motor neurons [11], projection neurons in layer 5 of the cerebral cortex [15], cerebellar Purkinje cells, or retinal photoreceptors [16]. In the spinal cord, even closely related interneuron populations were lost to different extents in *Pcdhg*^−/−^ animals, and the percent lost in these mutants was directly correlated with the percent gained in *Bax* null mutants in which developmental PCD was blocked [11]. This suggests that the γ-Pcdhs act as an upstream brake on PCD, but only in a subset of neuron types.

A targeted mutation removing the three γC isoforms within the *Pcdhg* cluster exhibited the same neonatal lethality and excessive PCD as *Pcdhg*^−/−^ mutants [17]. We used an unbiased CRISPR/Cas9 screen to further narrow this down to the γC4 isoform [18]. We found that mice with intact γC4 but lacking the other 21 γ-Pcdh isoforms (a mutant called *Pcdhg*^*1R1/1R1*^) survived to adulthood with nearly normal cell number in the spinal cord, while those lacking only γC4 with the other 21 γ-Pcdhs intact (a mutant called *Pcdhg*^*C4KO/C4KO*^) died shortly after birth with interneuron apoptosis in the spinal cord [18]. Further, we recently found that neuronal-restricted expression of γC4 from a Cre-inducible transgene was able to rescue postnatal viability in homozygous *Pcdhg*^*C4KO/C4KO*^ mutants, indicating that neuronal apoptosis is the cause of this early lethality [19]. In humans, biallelic variants in *PCDHGC4* cause a severe neurodevelopmental syndrome [20]. However, it has also been shown that mutation of the *Pcdha* and *Pcdhb* clusters in addition to *Pcdhg* exacerbates neuronal apoptosis, resulting in fewer viable neurons than loss of *Pcdhg* alone [8, 21]. Absence of P*cdha* or *Pcdhb* alone or together do not cause an increase in developmental apoptosis, suggesting that other isoforms may play a supporting role in neuronal survival that is largely compensated for in the presence of *Pcdhg*. This was most clearly demonstrated in the retina, where specific markers for diverse neuron types allow careful comparison of specific subtypes between mutants [21].

Here, we use a combination of *in vivo* and *in vitro* approaches to test a series of hypotheses about the role of the γC4 isoform in neuronal survival in the mouse retina. First, we compare *Pcdhg*^*C4KO*^ retinas to those from mice with expression disrupted from the whole *Pcdhg* cluster to ask if γC4 alone among gamma isoforms promotes survival in the retina. Second, we test if expression of *Pcdhgc4* corelates with the cell types that depend on γ-Pcdhs for survival. Third, we use live cell imaging of primary neurons cultured from *Pcdhg*^*C4KO/C4KO*^ retinas to test which domains of the protein are essential for its survival function.

## RESULTS

### *γ*C4 alone among gamma isoforms promotes neuronal survival in mouse amacrine cells

**Fig. 1:**
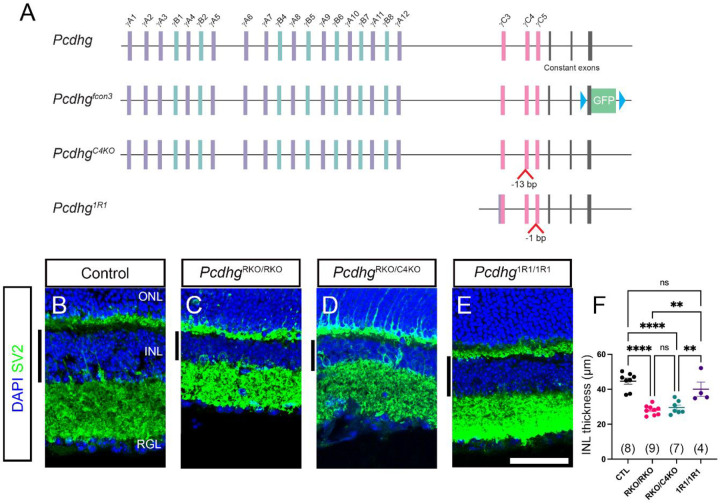
Thinning of the retinal inner nuclear layer in Pcdhg mutants. **A**) A schematic representation of the mouse *Pcdhg* locus and the three alleles used in this study: Variable exons are categorized as γA, γB, γC based on sequence homology while constant exons are common to all isoforms. The *Pcdhg*^*fcon3*^ allele includes loxp sites (blue triangles) flanking the GFP-fused third constant exon, and recombination of this allele disrupts all 22 isoforms. When crossed with *Pax6α-Cre*, the gene cluster is disrupted in the retina, which we refer to here as *Pcdhg*^*RKO*^. The Pcdhg^C4KO^ allele harbors a 13 bp deletion in the *Pcdhgc4* exon, resulting in a frame shift and loss of this isoform only. *Pcdhg*^*1R1*^ resulted from a large deletion spanning from *Pcdhga1* to *Pcdhgc3*, and a 1 bp deletion in *Pcdhgc5*. γC4 is the only isoform produced from this allele. **B**-**E**) Retina cryosections from the indicated genotypes were stained for DAPI and SV2 to clearly identify the major retinal layers. The thickness of the inner nuclear layer (INL, black bars to the left of each panel) was sampled across the length of the section and averaged to a single number for each retina. Each point in **F** represents a single retina and bars represent mean values. Genotypes were compared using ANOVA with Tukey post-hoc pairwise comparisons. Ns are indicated in parentheses in **F**. ** is p<0.01, **** is p<0.0001, ns is not significant. Scale bar is 50 μm.

The γ-Pcdhs in general, and the γC4 isoform in particular, are required for neuronal survival and postnatal viability in mice. However, α-Pcdh and β-Pcdh isoforms also support this function. We reasoned that, likewise, there may be other gamma isoforms that contribute to neuronal survival, but their roles may be masked by the massive apoptosis that ensues following loss of γC4. We began by testing if loss of γC4 alone will account for all the neuronal death that is seen in whole *Pcdhg* cluster knockouts. To do this we focused on the mouse retina by comparing three mutants; a conditional knockout resulting in loss of all γ-Pcdhs, *Pcdhg*^*fcon3*^ [11, 14], the *Pcdhg*^*C4KO*^ line lacking only γC4, and the *Pcdhg*^*1R1*^ mutant that retains intact γC4 but lacks all 21 other isoforms (Fig. 1A). To circumvent the neonatal lethality of *Pcdhg*^*C4KO*^ homozygotes, we generated compound heterozygotes also harboring one *Pcdhg*^*fcon3*^ allele and limited recombination to the retina by crossing to *Pax6α-Cre* [22]. *Pax6α-Cre* is expressed in progenitor cells giving rise to all types of neurons throughout the peripheral retina and specifically to Pax6-positive amacrine cells in the central retina. We and others have used this line previously to analyze *Pcdhg* functions in the retina [14, 23]. *Pax6α-Cre::Pcdhg*^*fcon3/fcon3*^ mutants are referred to here as Pcdhg^RKO/RKO^ for retina knockout and the compound heterozygous mutants as *Pcdhg*^*RKO/C4KO*^. Prior to Cre-mediated recombination, these latter mutants express *Pcdhgc4* only from the conditional allele; therefore, after Cre-mediated recombination, cells lack any expression of *Pcdhgc4* while expressing the other 21 *Pcdhg* isoforms from the *Pcdhg*^*C4KO*^ allele. If one of the other 21 isoforms contributes to survival, then *Pcdhg*^RKO/RKO^ should be more severely affected by neuronal apoptosis than *Pcdhg*RKO/C4KO.

**Fig. 2:**
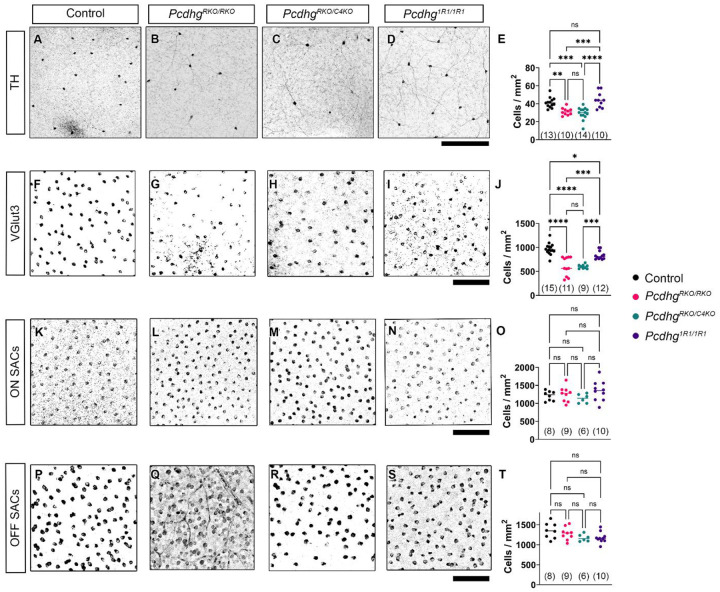
Loss of γC4 can account for the whole reduction in amacrine cells in *Pcdhg*^*RKO/RKO*^ mutants. Whole mount retinas from the indicated genotypes were stained for (**A**-**E**) tyrosine hydroxylase (TH) to label dopaminergic ACs, (**F**-**J**) VGlut3 to label glutamatergic ACs, and (**K**-**T**) Chat to label starburst ACs (SACs). SACs are analyzed separately as the ON subtype (**K**-**O**) and the OFF subtype (**P**-**T**) based on their layer position. Densities were calculated for each AC type, and genotypes were compared by ANOVA with Tukey post-hoc pairwise comparisons. Ns are indicated in parentheses on each graph. * is p,0.05, ** is p<0.01, *** is p<0.001, **** is p<0.0001, ns is not significant. Scale bar is Scale bar is 250 μm in **A**-**D**, 100 μm in other panels.

The retina is arranged in stereotyped layers of cell bodies and synapses. It consists of the outer nuclear layer (ONL) which contains photoreceptors, an inner nuclear layer (INL) where reside somas of amacrine cells (ACs), bipolar cells (BCs), and horizontal cells (HCs), and the retinal ganglion cell layer (RGL) which is comprised of retinal ganglion cells (RGCs) bodies and some populations of displaced ACs. These three layers are separated by two synaptic layers; the outer plexiform layer (OPL) and inner plexiform layer (IPL). The thickness of the cellular INL is particularly reduced by excessive cell death in *Pcdhg* mutants [14]. We first measured developmental apoptosis in these mice by measuring the thickness of the INL using DAPI staining in cryosections. We found that Pcdhg^RKO/RKO^ mutants had significant thinning in the INL compared with control or *Pcdhg*^*1R1/1R1*^mutants but were not different than *Pcdhg*^*RKO/C4KO*^ retinas (Fig. 1B–F). To investigate specific populations that undergo excessive developmental apoptosis in *Pcdhg* mutants, we assayed the density of three AC types in whole mount retinas: dopaminergic ACs labeled with tyrosine hydroxylase, glutamatergic ACs labeled with the vesicular glutamate transporter VGlut3, and cholinergic starburst amacrine cells (SACs) labeled with choline acetyltransferase (Chat; Fig. 2). SACs are composed of two subtypes identifiable by their location, with OFF-SAC cell bodies in the INL and ON-SAC cell bodies in the RGL. VGlut3+ ACs and TH+ ACs followed a similar pattern: Cell numbers were significantly reduced in Pcdhg^RKO/RKO^ retinas compared with controls but were not more severely affected than Pcdhg^RKO/C4KO^ mutants (Fig. 2A–J). Thus, among gamma isoforms, loss of γC4 alone can account for all the cell death from disruption of the whole cluster in these cell types. As we previously described in the spinal cord, cell number was not completely normal in *Pcdhg*^*1R1/1R1*^ retinas, as VGlut3+ ACs were significantly reduced compared with controls (although still significantly higher than Pcdhg^RKO/RKO^ or Pcdhg^RKO/C4KO^ animals). We suspect that this is because protein levels of γC4 are notably reduced in this mutant, albeit sufficient to prevent the massive neuronal apoptosis that leads to neonatal lethality [18]. TH+ ACs were not reduced in *Pcdhg*^*1R1/1R1*^ samples.

### Amacrine cell-type-dependence on γ-Pcdhs for survival correlates with expression of Pcdhgc4

**Fig. 3:**
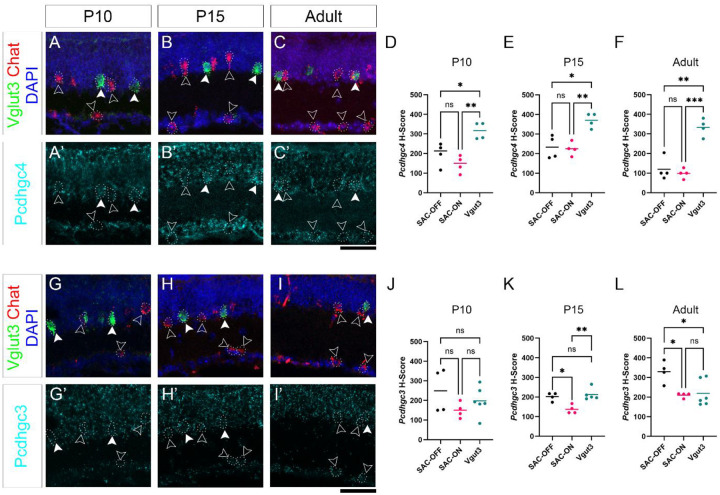
RNAScope analysis of *Pcdhgc4* and *Pcdhgc3* expression in SACs and VGlut3+ ACs. RNAscope was performed on cryosections from wild type retinas at the indicated ages for *Vglut3* (closed arrowheads), *Chat* (open arrowheads) and (**A**-**C**) *Pcdhgc4* or (**G**-**I**) *Pcdhgc3*. H-scores were calculated to approximate expression levels of both isoforms in each cell type (plotted in **D-F** for *Pcdhgc4*, **J-L** for *Pcdhgc3*). Means were compared by a Brown-Forsythe ANOVA and pairwise comparisons with Dunnett’s T3 multiple comparisons test. N=4 retinas per condition. * is p,0.05, ** is p<0.01, *** is p<0.001, ns is not significant. Scale bar is 50 μm.

In contrast to VGlut3+ ACs and TH+ ACs, SACs were not significantly reduced in any of our mutants (Fig. 2K–T). This is consistent with previous observation that SACs, the only cholinergic cell type within the retina, were largely preserved in the absence of *Pcdhg* [14]. It also parallels overall findings that only some neuron types undergo excessive apoptosis in *Pcdhg* mutants. Since γC4 is the major contributor to survival among gamma isoforms, we asked if this differential dependence was correlated with differential expression of *Pcdhgc4* during development. To do this we made use of RNAscope *in situ* hybridization (ISH) to analyze the expression of *Pcdhgc4* and *Pcdhgc3* (Fig. 3). We focused on comparing expression between Vglut3+ ACs and SACs, as both are from the amacrine lineage and have similar densities and distributions in the retina. To analyze expression, we calculated an H-Scores for each retina, which is a weighted estimation of ISH dots present in each neuron. This method is semi-quantitative, assigning each cell to a bin valued from 0 (no expression) to 4 (high expression) then multiplying the percent number of cells in each bin by its value to calculate a single number between 0 and 400 representing the expression of each target transcript (*Pcdhgc4* or *Pcdhgc3*). We chose time points to capture the peak of developmental apoptosis of amacrine cells and the peak of excessive apoptosis in *Pcdhg* mutants and beyond [14, 24]. VGlut3 ACs were not detectable by RNAscope at P5 but were by P10. Thus, we analyzed expression in P10, P15, and adult retinas. Using this method, we found that throughout development, Vglut3+ amacrine cells expressed significantly more *Pcdhgc4* mRNA than did ON or OFF SACs (Fig. 3A–F). In contrast, the expression of *Pcdhgc3* mRNA was more varied over time: At P10 there were no differences between the three types, at P15 ON-SACs expressed slightly less *Pcdhgc3* than the other two types, and in adults OFF-SACs expressed slightly more *Pcdhgc3* than the other types (Fig. 3G–L).

**Fig. 4:**
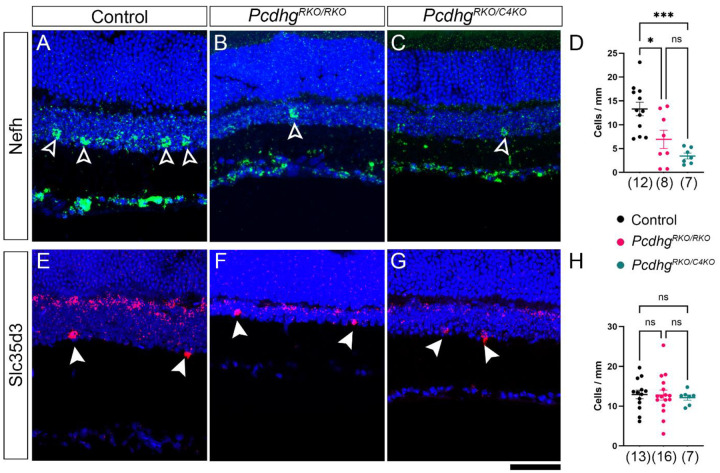
*Slc35d3*+ and *Nefh*+ ACs have differential dependence on *Pcdhg* for survival. RNAScope was used to label (**A-D**) *Nefh*+ and (**E**-**G**) *Slc35d3*+ ACs in the indicated genotypes. Cells were counted and normalized to the length of the section to estimate density (**D**,**H**). Genotypes were compared by ANOVA with Tukey post-hoc pairwise comparisons. Ns are indicated in parentheses on each graph. * is p,0.05, *** is p<0.001, ns is not significant. Scale bar is 50 μm.

**Fig. 5:**
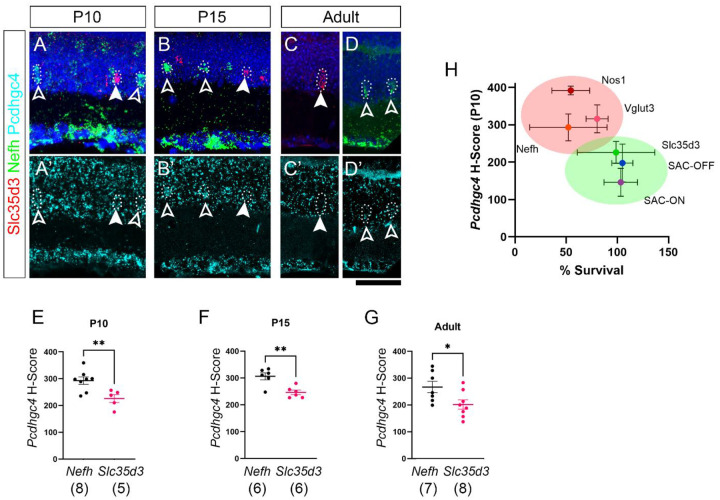
RNAScope analysis of *Pcdhgc4* expression in *Slc35d3*+ and *Nefh*+ ACs. **A**-**D**) RNAscope was performed on cryosections from wild type retinas at the indicated ages for *Slc35d3* (red, closed arrowheads), *Nefh* (green, open arrowheads) and *Pcdhgc4* (Cyan, lower panels). H-scores were calculated to approximate expression levels in both cell types (plotted in **E**-**G**). Means were compared by student’s t-test. Ns are indicated in parentheses on each graph. * is p,0.05, ** is p<0.01, *** is p<0.001, ns is not significant. Scale bar is 50 μm. **H**) The mean H-score for *Pcdhgc4* at P10 in 6 AC types is plotted on the y-axis with the percent survival in *Pcdhg*^*RKO/RKO*^ on the x-axis. Error bars indicate standard deviation in x and y. Cell types with near total survival are encircled in the green oval, while types with significant cell loss are encircled in the red oval.

The comparison of SACs and VGlut3+ ACs supports the hypothesis that expression of *Pcdhgc4* correlates with a cell type’s reliance on γC4 for survival. To test this further, we sought another AC type that remained largely preserved in *Pcdhg* mutants. To do this we made use of available single-cell RNAseq data from amacrine cell types to identify candidate markers [25]. We chose four markers, *Slc35d3*, *Nefh*, *Tpbg*, and *Cbfa2t3*, and analyzed their distribution in retinal cryosection by RNAscope. Two of these – *Slc35d3* and *Nefh* – met our criteria of labeling discrete, countable cells and having an even distribution along the length of any given retina cryosection. To limit our analysis to ACs, we focused on cell bodies located in the amacrine portion of the INL (i.e., the portion of INL closest to the IPL, as the outer half of INL contains a higher percentage of BCs and HCs). This meant excluding any ACs displaced to the RGL, which was of particular importance with *Nefh* as it is expressed by RGCs. *Slc35d3* also labeled a population of cells in the portion of the INL adjacent to the OPL, which could be BCs or HCs but were excluded from our analyses. To estimate the density of these cells, we collected images from entire retina cryosections, counted manually, and normalized to the total length of retina in the section. We found that *Nefh*+ ACs were significantly reduced in both *Pcdhg*^*RKO/RKO*^ and *Pcdhg*^*RKO/C4KO*^ mutants. Consistent with our analyses of VGlut3+ ACs and TH+ ACs, *Pcdhg*^*RKO/RKO*^ were not more severely affected than *Pcdhg*^*RKO/C4KO*^ retinas (Fig. 4A–D). Conversely, when *Slc35d3*+ ACs were counted, there were no reductions in either mutant (Fig. 4E–H). To test if this differential dependence on γ-Pcdhs for survival also correlated with differential expression of *Pcdhgc4*, we repeated RNAscope analyses using these cell types at the same time points analyzed previously, finding that *Slc35d3*+ ACs consistently expressed lower levels of *Pcdhgc4* than did *Nefh*+ ACs (Fig. 5A–G). To visualize this correlation, we plotted H-scores for *Pcdhgc4* for each cell type at P10 by the percent cell loss for that type in Pcdhg^RKO/RKO^ mutants, including analysis of AC cells brightly positive for Nos1 in the INL (Fig. 5H). The cell types clearly separated into two groups: those with high H-score and low survival in *Pcdhg*^*RKO/RKO*^ mutants in the upper left quadrant and those with low H-score and high survival in *Pcdhg*^*RKO/RKO*^ mutants in the lower right quadrant. Based on these data, we conclude that for mouse amacrine cells, expression of *Pcdhgc4* does correlate with cell type reliance on γC4 for survival.

### γ*C4 promotes neuronal survival through its variable cytoplasmic domain*

**Fig. 6:**
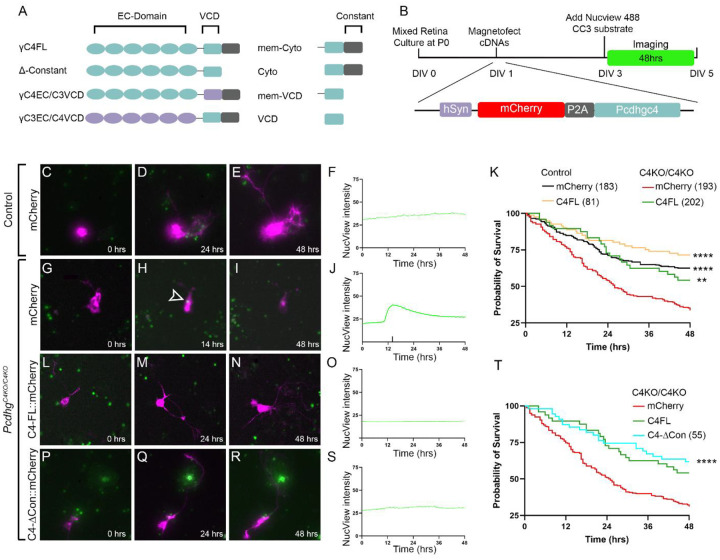
Long-term live cell imaging of γC4 promotion of neuronal survival. **A**) A schematic of the truncation and domain swap constructs used in the live cell imaging experiments. **B**) The experimental time course with a schematic of the bicistronic rescue constructs. Mixed retinal neurons cultured at P0 from (**C**-**F**) control or (**G**-**R**) *Pcdhg*^*C4KO/C4KO*^ retinas were transfected with the (**C**-**J**) mCherry, (**L**-**O**) mCherry and full-length *Pcdhgc4* cDNA, or (**P**-**S**) mCherry and truncated *Pcdhgc4* cDNA without a constant domain. Images of transfected neurons were collected for 48 hours at 30-minute intervals in the presence of NucView 488. A spike in the green NucView signal (e.g., **J**) indicated apoptosis, and was used to measure survival time (plotted in **K**, **T**). To test for rescue, survival in each condition was compared to matched (i.e., within the same 24-well plate) *Pcdhg*^*C4KO/C4KO*^ neurons transfected with mCherry by log-rank Mandel-Cox test. ** is p<0.01, **** is p<0.0001, scale bar is 50 μm. Ns are the number of neurons imaged per condition and are indicated in parentheses. C4FL and mCherry data from **K** are replotted in **T** for comparison.

Each γ-Pcdh isoform is modularly similar with six extracellular cadherin EC repeats and a common cytoplasmic C-terminus separated by a transmembrane domain and a membrane-proximal variable cytoplasmic domain (Fig. 6A). The observation that γC4 alone is specialized for the unique function of neuronal survival raises the question of which protein domain is essential for this action. Extracellularly, each isoform engages in preferentially homophilic interactions in *trans* but can have a variety of interactors in *cis*, including other cPcdh isoforms and other cell adhesion molecules such as neuroligins [26–30]. Isoform-specific interactors have also been described for the cytoplasmic domains, including GABA_A_R (for γC5) and Axin1 (for γC3) [31–33].

To test which γC4 domains are required for its survival function, we turned to an *in vitro* approach amenable to manipulation. We cultured mixed retinal neurons collected from P0 pups in the hours before *Pcdhg*^C4KO/C4KO^ animals normally die. Cultures were maintained with serum for two days before switching to serum-free neurobasal media. After four days in neurobasal media, cultures were fixed and analyzed for cleaved caspase-3, a marker of apoptosis. Apoptosis was observed in both control and mutant cultures but was more notable in neurons from *Pcdhg*^C4KO/C4KO^ animals. We chose a rescue approach based on transfection to test specific domains, but found that endpoint analyses were limited as transfection efficiency could vary between plasmid DNA constructs and neurons that underwent apoptosis early in the culture could be barely detectable at the endpoint. To overcome these limitations, we used long-term live cell imaging to follow cells prospectively as they progressed in culture (Fig. 6B). As before, *Pcdhg*^C4KO/C4KO^ animals and their control littermates were cultured at P0. At one day *in vitro* (1 DIV) cultures were transfected by magnetofection with a series of *Pcdhgc4* cDNA constructs encoding distinct combinations of protein domains, each with a C-terminal FLAG tag (Fig. 6A). Each construct was designed with a synapsin promoter to limit expression to neurons, and mCherry and *Pcdhgc4* transgenes were separated by a P2A bicistronic linker. We verified in separate cultures that each mCherry-positive cell also expressed the linked *Pcdhgc4* variant by staining for the FLAG tag. Forty-eight hours after transfection, media was changed to neurobasal media including the cell membrane-permeable reporter NucView 488, which fluoresces brightly green after caspase-mediated cleavage. Cells positive for mCherry were identified and imaged over the following 48 hours using a Lumascope microscope within a tissue culture incubator. Approximately 50 regions of interest (ROIs) per culture were imaged at 30-minute intervals. Images were analyzed by measuring intensity of the green (apoptotic) signal within the cell body of mCherry-positive neurons, and the time of apoptosis was identified by a spike in the green signal (e.g., Fig. 6J). This time of apoptosis was used to plot neuron survival over the 48-hour imaging period. Comparing neurons from *Pcdhg*^C4KO/C4KO^ and control retinas transfected with mCherry verified the observation of excessive apoptosis in these cultures (Fig. 6C–K, **Supplemental Video 1 and 2**). We then moved to testing if full-length *Pcdhgc4* could rescue apoptosis in our system. The survival rates for *Pcdhg*^C4KO/C4KO^ neurons transfected with the full-length *Pcdhgc4* construct were indistinguishable from control neurons, while expression of *Pcdhgc4* in controls had no discernable effect on survival (Fig. 6K–O, **Supplemental Video 3 and 4**).

**Fig. 7:**
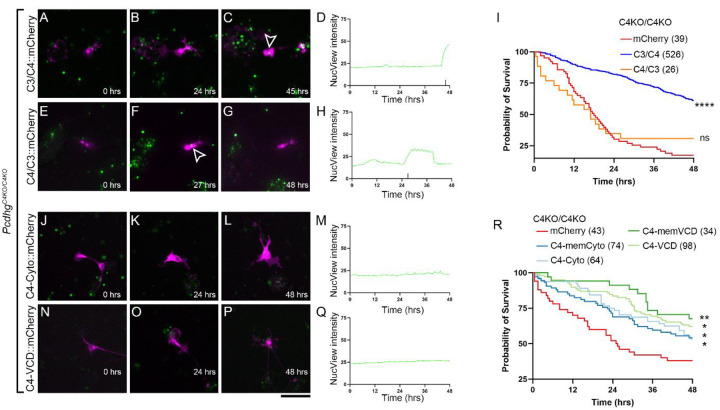
γC4 promotes neuronal survival through its variable cytoplasmic domain. Live cell imaging of *Pcdhg*^*C4KO/C4KO*^ neurons transfected with (**A**-**D**) a C3/C4 construct comprised of the extracellular domain of γC3 and the cytoplasmic domain of γC4 and (**E**-**H**) the converse C4/C3 construct with the extracellular domain of γC4 and the intracellular domain of γC3. **I**) Survival analysis demonstrates that the cytoplasmic domain of γC4 is essential to prevent apoptosis, while the extracellular domain is not. Neurons transfected with (**J**-**M**) the entire cytoplasmic domain only or (**N**-**Q**) the variable cytoplasmic domain only (excluding the constant domain) also rescued cell death (**R**). Membrane-targeting of these domains was not required. * is p<0.05, ** is p<0.01, **** is p<0.0001, ns is not significant, by log-rank Mandel-Cox test compared with matched *Pcdhg*^*C4KO/C4KO*^ neurons transfected with mCherry. Scale bar is 50 μm. Ns are the number of neurons imaged per condition and are indicated in parentheses.

As our assay was sensitive to detect γC4-mediated promotion of neuronal survival, we began to test specific domains. The C-terminal constant domain is common to all γ-Pcdh isoforms, but it could still conceivably be required for the survival function of γC4 in combination with its other, unique domains. We tested a γC4-ΔCon construct lacking the entire constant domain and found that it was able to rescue survival as effectively as the full-length construct (Fig. 6P–T, **Supplemental Video 5**), indicating that, as expected, constant domain interactions are not required for γC4’s unique function. To test the importance of intracellular and cytoplasmic interactions, we made a pair of constructs swapping domains with γC3, which is not required for neuronal survival [32]. The C3/C4 construct encoded the extracellular domain and transmembrane domain of γC3 with the cytoplasmic domain of γC4, while C4/C3 was the converse – the extracellular domain and transmembrane domain of γC4 with the cytoplasmic domain of γC3 (Fig. 6A). We found that C3/C4 completely rescued survival, while C4/C3 expression had no effect, with apoptosis being indistinguishable from that of cells transfected with mCherry alone (Fig. 7A–I, **Supplemental Video 6 and 7**). This indicates that extracellular interactions are not required for γC4-mediated survival, but rather suggests that intracellular interactions mediated by the unique γC4 variable cytoplasmic domain are essential. We then asked if these interactions are sufficient for this function, and if they require any extracellular domain or even localization to the plasma membrane. For this, we tested a series of cytoplasmic only constructs, encoding either the variable cytoplasmic domain only (VCD) or the VCD with constant domain (cyto) with or without a tag to localize the protein to the plasma membrane. We found that all four of these constructs could rescue compared with mCherry alone, with the VCD constructs slightly more effective than the entire cytoplasmic domain (Fig. 7J–R, **Supplemental Video 8–11**). Membrane tethering had no significant effect on survival. Thus, the VCD of γC4 is necessary and sufficient to promote neuronal survival independent of specific localization to the cell membrane.

## DISCUSSION

Here we present three main conclusions: 1) Cell death of retinal amacrine cells resulting from loss of all γ-Pcdhs can be matched by loss of γC4 only; 2) cell type dependence on γ-Pcdhs for survival correlates with expression of the *Pcdhgc4*, coding for the γC4 isoform; 3) the variable cytoplasmic domain of γC4 is necessary and sufficient for its pro-survival function.

We found previously that γC4 was essential for neuronal survival in the spinal cord [18], which was confirmed for interneurons of the cerebral cortex [34], but there is also strong support for a role for other isoforms in promoting survival. Hasegawa and colleagues made large, multi-cluster deletions including disruption of all three cPcdh loci. Only deletions that included *Pcdhg* exhibited excessive apoptosis, but the triple cluster knockout (*Pcdha*, *Pcdhb*, and *Pcdhg*) was the most severely affected with significantly more cell death than in the *Pcdhg* mutant alone [8]. More analogous to our current study, Ing-Esteves and colleagues targeted the constant exons of *Pcdha* on the *Pcdhg*^*fcon3*^ allelic background, making a conditional allele with disrupted alpha and gamma isoforms in Cre-expressing cells. They used this mouse line to analyze retinal phenotypes including cell death, and described substantially more cell loss in the double-cluster mutant than in *Pcdhg* mutants alone [21]. That study included an elegant gene dosage analysis that found increasing apoptosis with decreasing copies of *Pcdha* in animals without *Pcdhg*. Together, these findings suggest two possible explanations. In the first hypothesis, γC4 is the major isoform that promotes neuronal survival, but *any* other cPcdh isoform can partially compensate for its absence. Decreasing gene dosage of α-Pcdhs decreases the overall level of cPcdh and likewise the ability to compensate for survival. In the second hypothesis, γC4 is the major isoform that promotes neuronal survival, but some other *specific* isoform can partially compensate for its absence. Our findings are more consistent with this second hypothesis, as the isoform dosage difference between *Pcdhg*^*RKO/C4KO*^ and *Pcdhg*^*RKO/RKO*^ (21 isoforms) is greater than the total number of isoforms in the alpha cluster (14 isoforms). Growing evidence has pointed to the prominence of the C-type isoforms [32, 35–37], suggesting the hypothesis that one of the αC isoforms could be responsible for partially compensating for loss of γC4. αC2 would seem a particularly attractive candidate, as it and γC4 are the only isoforms constrained in humans [18].

Our finding that expression of *Pcdhgc4* is higher in cell types that depend on this isoform for survival builds on a recent report from Mancia Leon and colleagues that analyzed isoform expression in the cerebral cortex [34]. They found that *Pcdhgc4* was highly expressed in GABAergic neurons in the visual cortex (which undergo excessive apoptosis in *Pcdhg* mutants [10, 12]), but was not prominently expressed in glutamatergic neurons which survive in normal numbers without the γ-Pcdhs [15]. Our comparison was between cell types from the amacrine lineage which arise in the same location – unlike GABAergic and glutamatergic projection neurons in the cortex, which arise in distinct areas of the subventricular zone and then intermingle via migration. Based on single-cell analyses of Purkinje neurons, it was originally thought that all neurons express the C-type isoforms while a handful of the other isoforms were expressed in a stochastic manner within each cell [38–40]. Later studies identified the mechanisms of isoform expression choice within a cell, including methylation of each gene’s promoter region and alterations in chromatin structure [41, 42] and the processivity of the cohesin complex [43]. Our findings and those of Mancia Leon and colleagues suggest that expression of *Pcdhgc4* may be regulated via a distinct mechanism. The observation from the spinal cord that excessive cell death in *Pcdhg* null neurons correlates with the extent of normal developmental apoptosis [11] suggests that this *Pcdhgc4*-specific gene regulation could be connected with the larger cell-type-specific program of developmental cell death.

Our final major finding here is that γC4 promotes neuronal survival through its variable cytoplasmic domain. This is the domain that is most divergent between isoforms and the site of other known isoform-specific interactions such as that between Axin1 and γC3 [31]. Kobayashi and colleagues found that a very large deletion removing the entire *Pcdha*, *Pcdhb*, an *Pcdhg* cluster up to but not including the γC isoforms resulted in widespread neuronal apoptosis and neonatal lethality [44]. This prompted the hypothesis that γC4 might require *cis* heterodimerization with other isoforms to promote survival. *Cis* dimerization requires EC domain 6 in the extracellular region [45, 46]. Indeed, γC4 is unique among gamma isoforms in that it cannot efficiently traffic to the plasma membrane in heterologous cells without a “carrier” isoform due to its EC6 sequence [29, 47]. At least in our transfection-based system, the VCD alone was sufficient to promote neuronal survival and did not require the context of a larger cPcdh complex. Further, fusing the γC4 cytoplasmic domain with the γC3 extracellular domain did not reduce its efficacy. Thus, these unique features of the γC4 extracellular domain are not required for its role in neuronal survival. The observation that VCD alone can rescue survival also provides evidence against the hypothesis that excessive cell death is secondary to any function in directing circuit formation.

The findings here are part of an emerging understanding of the roles of cPcdh isoform diversity and roles for specific isoforms. γC3 is essential for dendrite arborization in the cerebral cortex and for synaptic connections in the dorsal spinal cord [32, 35]. γC5 appears to have a number of unique functions related to Alzheimer’s pathogenesis [36, 48, 49]. αC2 particularly promotes serotonergic axon dispersion [37, 50]. Other functions like self-avoidance in SACs and dispersion of primary sensory axons in skin require isoform diversity [35, 51]. It has not been determined if γC4 can also contribute to these processes.

## METHODS

### Mouse Strains

All mouse lines were housed at the animal research facilities of Wayne State University under standard housing conditions, where mice were provided with provided food and water ad libitum and maintained under a 12hr light/12 hr dark rhythm. All procedures were performed in accordance with The Guide for the Care and Use of Laboratory Animals and were approved by the Institutional Animal Care and Use Committee at Wayne State University. Both male and female mice were used in the study. The following previously described mouse lines were included in this work: Pax6α-Cre [22]; *Pcdhg*^fcon3^ [11]; *Pcdhg*^C4KO^; and *Pcdhg*^1R1/1R1^ [18]. For simplicity, *Pax6α-Cre* crossed with *Pcdhg*^*fcon3*^ is referred to here as *Pcdhg*^*RKO*^ for retina knock-out.

### Immunofluorescence

To prepare whole mount retinas, adult animals > P42 were sacrificed via CO_2_ asphyxiation and cervical dislocation. Eyes were enucleated and dissected to remove both cornea and lens; the remaining eye cups were fixed in 4% paraformaldehyde (PFA) in PBS at 4°C for 3 hours. After fixation, the retina was dissected from RPE and incubated free-floating in primary antibody and blocking solution of 2.5% Bovine serum albumin with 0.1% Triton-X-100 in PBS for 48hrs at 4°C. Retinas were then washed in PBS and incubated with an Alexa-Fluor conjugated secondary antibody in the same blocking buffer overnight at 4°C. After extensive washing in PBS, retinas were mounted on slides in 80% glycerol.

To prepare retinal cryosections, after 4% PFA fixation eye cups were cryoprotected in 30% sucrose, then embedded in optimal cutting temperature (O.C.T) compound (Sakura-Finetek). Retinas were sectioned at 12 μm onto positively charged Superfrost Plus slides (Fisher Scientific) in a cryostat. The resulting tissue slides were blocked with 2.5% bovine serum albumin as above for 1 hour and then incubated in primary antibody in blocking buffer overnight at 4°C in a humidified chamber. The next day slides were incubated with secondary antibodies in PBS for 1 hour at room temperature. Tissue sections were counterstained with DAPI and mounted with Fluoromont G mounting media (Invitrogen).

### Antibodies

Primary antibodies used in this study include: goat anti-Chat (1:500, Millipore, RRID:AB_2079751), rabbit anti-Nos1 (1:500, Sigma RRID:AB_260796), Mouse anti-SV2 (1:50, Developmental Studies Hybridoma Bank, RRID:AB_2315387), sheep anti-Tyrosine Hydroxylase (1:500, Millipore, RRID:AB_11213126), Guinea pig anti-Vglut3 (1:10,000, Millipore, RRID:AB_2187832). Secondary antibodies were Alexa Fluor conjugated 488, 594, or 647 (1:500, Invitrogen).

### RNAscope in situ hybridization

Eyes were collected for RNAscope at P5, P10, P15 and Adult. RNAscope (ACDbio) was performed following the manufacturer’s protocol with the RNAscope Multiplex Fluorescent Assay v2 for fixed-frozen tissue. Retinas were sectioned at 12 um for all assays. Tissue from control and mutant animals were mounted on the same slide where appropriate to minimize variability in processing. Tissue slides were immersed in target retrieval reagent for 5 minutes. Where co-detection with cleaved caspase antibody was required, the RNAscope Multiplex Fluorescent Assay v2 co-detection for fixed frozen tissue protocol was followed, again with only a 5-minute incubation in co-detection target retrieval reagent. Probes used for RNAscope included *Pcdhgc4* (ACD 835791-C3), *Pcdhgc3* (ACD 802841), *Chat* (ACD 408731 and 408731-C3), *Vglut3* (*Slc17a8*, ACD 431261-C2), *Nefh* (ACD 443671-C2), *Slc35d3* (ACD 545921), *Tpbg* (ACD 425681) and *Cbfa2t3* (ACD 434601-C2). TSA vivid dyes were used at a concentration of 1:750. For co-detection cleaved caspase 3 primary antibody was used at 1:400.

### Image Acquisition and Quantification:

Whole mount retinas were imaged for cell density using a Leica SP8 confocal microscope and Leica Application Software (LASX) to capture z stacks from two to three positions per retina located midway between the central and peripheral retina. To determine the density of amacrine cell types, image stacks were analyzed using the Cell Counter plugin of Fiji image analysis software [52]. Multiple images from the same retina were averaged and retinas were used as biological replicates. Neuronal densites were compared between genotypes using one-way ANOVA and Tukey’s post hoc test.

To determine the expression level of *Pcdhgc4* and *Pcdhgc3* over development, the number of RNAscope ISH dots were quantified using Qupath Software [53]. Cell bodies were identified using the Cell Detection feature, then the number of dots per cell body were estimated using the Subcellular Detection feature of Qupath. Thresholds for each probe remained constant per imaging session. Expression is reported as an H-score according to the manufacturer’s recommendation (ACDbio). Briefly, each cell was given a value based on the number of ISH dots, 0 (for 0 ISH dots), 1 (1–3 dots), 2 (4–9 dots), 3 (10–15 dots) or 4 (more than 15 dots). The percentage of cells in each bin was multiplied by the bin value to generate a single number between 0 and 400. Statistical significance was determined using Wilcoxon Rank Sum test in R.

To compare the thickness of the inner nuclear layer between genotypes, retina sections were imaged on a Leica Fluorescent microscope. Using the measure function of Fiji software the thickness of the INL was measured at four points evenly spaced across the cell layer and then averaged. Statistical significance was determined using one-way Anova and Tukey’s post hoc test.

Cell counting to determine *Nefh-* and *Slc35d-* and *Nos1-* positive AC densities were performed in whole retina sections using a Leica Fluorescent microscope. Cells were counted manually and normalized to the length of the section. Means per retina were compared across genotypes using one-way ANOVA and Tukey’s post hoc test.

### cDNA Constructs

Protocadherin-γC4 full-length, truncation, and domain-swap (with γC3) constructs were subcloned from previously describe constructs [28] and by direct synthesis (IDT). The vector began as pCAG and the CAG promoter was exchanged with a minimal human synapsin promoter (hSyn). Coding sequence for a membrane targeted mCherry was introduced downstream of hSyn, followed by a P2A bicistronic sequence. *Pcdhg* cDNAs were subcloned in frame and 3’ to mCherry at a NotI site. All assembly was done using NEBuilder Hifi Assembly (New England Biolabs).

### In vitro survival rescue assay

Mixed retina cultures were prepared with some modifications as described [54, 55]. Briefly, *Pcdhg*^C4KO/C4KO^ pups and their control littermates were euthanized and enucleated at P0. Retinas were dissected out of the eye cup and submerged in 10 units/mL papain solution (Worthington) with DNAse for 20 minutes at 37°C. Retinas were transferred sequentially to a light and heavy inhibitor solution made of BSA and trypsin inhibitor to inactivate papain before disassociation. Retinas were then dissociated in a plating media of 2.5% FBS, 1% Pen/Strep and 1% N2 supplement. Dissociated retina was plated at a density of 50,000 cells/well of a glass-like bottom 24 well plate (Cellvis) coated in poly-lysine, where each row of the plate contains cells from one animal. At DIV 1, cultures were transfected with 1ug of cDNA per well using Neuromag (1:1 Oz Biosciences) according to the manufacture’s protocol with three cDNA constructs per plate. Approximately two hours post transfection, plating media was replaced, and cultures maintained until DIV 3 when plating media was replaced with a modified retina growth medium and Nucview cleaved caspase substrate-488 as described [54, 56]. Immediately after media change the culture plate was moved to a Lumascope LS850 (Etaluma) for live-cell imaging. Two ROIs with mCherry positive neurons were identified per well using the Lumaview Pro software. A protocol for the imaging session was prepared so that each ROI was captured for both mCherry in red (presence of cDNA construct) and Nucview-488 in green every 30 minutes for 48 hours. Analysis of the live imaging time course was performed by marking the time (hrs) an mCherry positive neuron had a spike in Nucview-488 signal indicating active apoptosis in transfected neurons. All mCherry neurons per ROI were counted and the total number of neurons assayed varied with transfection efficiency. To test for rescue, survival curves for each construct were compared with matched *Pcdhg*^C4KO/C4KO^ neurons from the same culture plate transfected with mCherry alone by log-rank Mandel-Cox test. Survival curves and statistics were generated using simple survival analysis (Kaplan-Meier) from GraphPad Prism v10.2.3.

## Data Availability

All supporting data are available upon request to the corresponding author.
